# Periscope: Surgery

**Published:** 1818-07-01

**Authors:** 


### Surgery.

1. *Bronchotomy.*—The importance of laryngeal diseases, and the dreadful consequences which frequently result from inflammation about the glottis, have lately pressed themselves upon our notice. But the most interesting case ever published, and which indeed stands *unique* in the annals of Surgery, is that recorded in the *Medico-Chirurgical Journal* for January; where a tube has continued for more than a year to perform the vicarious office of a rima glottidis. The case is briefly this—A gentleman was seized with laryngeal inflammation from exposure to cold; and, notwithstanding that the most decisive evacuations, general and local, with counter-irritants, &c. were employed, the inflammation ran so high that the unfortunate gentleman was on the very point of strangulation, when Drs. Denmark and Johnson proceeded to the operation of Bronchotomy. Such was the violent action, however, in the long muscles of the neck, and such the tumefaction of the integuments from blisters and sinapisms, that the operation was extremely difficult. At length the trachea was laid bare, close under the thyroid gland, and a tube introduced. Days and weeks passed away, but the tube could not be dispensed with. Nearly two months after the operation, several pieces of calcareous deposition that had formed a part of the walls of the larynx came away through the tube. His health became good; but to this day (June, 1818), nineteen months from the date of the operation, he breathes by the tube. The wound gives little trouble. The tube is taken out and cleaned twice a week, and immediately replaced. When he wishes to speak, he closes the end of the tube with his finger, and forces enough of air through the natural passage for the pronounciation of his words.

2. *Wounds.* In the same Journal, Dr. Boyd records some interesting cases of amputation on board the Gorgon from bad gunshot wounds; and after inflammation, tumefaction, and symptomatic fever, had been succeeded by profuse suppuration. One at the shoulder joint succeeded; but three of the thigh, near the trochanter minor, proved fatal.

Several cases of *Tetanus* supervening on wounds, occurred in the squadron; but only one on board the Gorgon, which Dr. Boyd attributes, in a good measure, to the great attention that was paid in the latter ship to *intestinal evacuations*, during the whole period of the wounded mens' confinement. In this we fully agree with Dr. Boyd, and also with Dr. Dickson, who was then, we believe, physician on the expedition.

The wounded were treated by Dr. B. on strict antiphlogistic principles. Blood was abstracted from the system, whenever inflammation, pain, and tension presented themselves; laxatives were given; low diet was enjoined; and the injured parts were kept constantly wet with cold lotions. Warm applications were injurious, except where tension prevailed, without morbid heat, and a disposition to gangrene. Here warm fomentations, opiates, and antimonials were serviceable.

*Sloughing.* A strong disposition to sloughing occurred among several of the wounded, demanding antiphlogistic measures of great rigour. " I am persuaded," says Dr. B. " that many a patient has fallen a victim to the premature exhibition of bark, wine, opium, and brandy, when a dark-coloured speck has appeared on the surface of a wound, or of an inflamed tumour. In such case, therefore, in my opinion, the primary and more early treatment ought to be invariably, and *in all its parts*, anti-inflammatory.

3. *Necrosis of the tibia in a boy.* In a boy, whose thigh was amputated by Dr. Johnson, the knee joint was found completely dislocated and anchylosed. The integuments of the amputated thigh were precisely like brawn, and no trace of muscular fibre could be seen. Yet the wound healed by the first intention. The whole tibia was dead, and surrounded by a beautiful specimen of new bone, in all stages of secretion. The vascularity of the periosteum, in some places, was astonishing.

4. *Tense elastic tumour on the patella.* We frequently meet with these, and find them very obstinate. Mr. Simon, of Chester, [*Repos. No.* 49.] relates a case of this kind which baffled every remedial measure, till he laid it open, when it was found to contain nothing but blood. The wound healed readily.

5. *Syphilis.* In the original department we entered our caveat against the new doctrine of trusting the cure of syphilis to sarsaparilla, in exclusion of mercury. The paper of Dr. Thompson, in the 53d No. of the *Ed. Journal,* supported by the instructions issued from the Army Med. Department will give the fullest scope to the new practice, and will either do much good or much harm. Dr. Thompson has been led to the same conclusion as some of the older writers, namely, that sarsaparilla is possessed of *singular efficacy* in the cure of symptoms usually reputed syphilitic. In the various forms of syphilis remaining after, or succeeding to, the use of mercury, such as cutaneous eruptions and ulcerations, ulcerations of the throat, pains and swellings of the joints and ligaments, and nodes of the bones, he has found the simple decoction of sarsaparilla efficacious, with attention to regimen, and proper local treatment.

From this Dr. T. advanced one step farther, and treated, at the Depôt Hospital, in Edinburgh, both primary and secondary syphilitic symptoms without mercury, and only enforcing an antiphlogistic regimen and horizontal position, with mild local applications. Under this treatment chancres and buboes have, in every instance, disappeared, as speedily as where mercury is employed.

Dr. T. remarks, that "Bubo in one or both groins has occurred in about *one-fourth* of those affected with chancre." Now from very ample experience in syphilitic complaints, we think we may safely affirm that this proportion of buboes supervening on chancre far exceeds that where mercury is properly administered. Dr. T. says that, under the new practice, the disposition to gangrenous inflammation, or to phagedenic ulceration, "so *common* in the treatment of these affections under the *most careful* employment of mercury," did not manifest itself. Here again we beg to state, that from very long and very extensive clinical observation, we are authorized to aver, that these phagedenic and gangrenous dispositions, so far from being *common* occurrences under *careful* administrations of mercury, and proper rest and abstinence, are extremely rare; not more, probably, than one case in one hundred. Indeed these occurrences are almost always, even in *mali habitus,* ocasioned by want of attention to venesection, purgatives, abstinence, and rest, which ought to be invariably employed to lower the tone of the constitution, and adapt it to the more speedy action of the mineral. This then is no solid argument in favour of the new practice.

Dr. T. observes that a *hard tubercle,* accompanied by discoloration of the skin, has remained for a considerable time after cicatrization, frequently shewing a disposition to ulceration, when neglected or irritated. Surely this is a suspicious circumstance. In fact, we have long observed in our own practice, that this hardness remaining after the healing of a chancre, was too often premonitory of secondary symptoms; and for many years we have not considered the constitution safe, till this induration was dispersed under the mercurial influence. But again, " A sufficient time, says Dr. T. has not yet elapsed to enable us to ascertain in how many cases constitutional affections will occur, or what all the constitutional may be among those who have been cured of the *primary* symptoms of syphilis without the use of mercury." It certainly is not a very flattering omen of success to the new practice to find *one in ten* of the patients thus treated become affected with *secondary* symptoms. Surely Dr. Thomson must be a little warped by prejudice, when he says that this is not a greater proportion than where mercury is used. After nearly thirty years experience, we have no hesitation in asserting that it is, at the very least, *treble* the proportion which has occurred in our own practice. And, moreover, we venture to affirm that, a supervention of *secondary* symptoms in every tenth case, would ruin the character of any *private Practitioner,* who had a character to lose. Let the latter class then, for whose use this Journal is principally designed, pause ere they hazard their own reputation, and their patients' lives, by the indiscriminate rejection of mercury from their list of antisyphilitic remedies. Let them remember the fate of *Nitric Acid!* Let them, however, not run into the opposite extreme of salivating their patients for every excoriation or syphiloid symptom. Let them study the *first* article in this Journal, and they will ultimately find that—

In Medio tutissimi ibunt.

*Injury of the Head.* Mr. Cunningham, of the Royal Navy, relates [*Med. Chir. Jour.* 26], a remarkable instance of the difficulty of ascertaining the seat and nature of cerebral lesions from blows on the head. A seaman fell down a hatchway and pitched on his head; was taken up insensible, carefully examined, and only a slight contusion could be discovered on the right *os mali.* But remaining comatose, with contracted pupils, he was bled largely without relief. Stimulants were then tried with similar success. The head being shaved, and nothing found to indicate the seat of fracture, a perpendicular incision was made through the right temporal muscle down to the zigoma of the *os mali,* and another across the *os frontis* down to the bone; but no fissure or fracture was discovered. Things continuing desperate, the trephine was applied to the right side of the *os frontis,* close to the coronal suture. No blood nor other fluid was found, and the coverings of the brain were sound. He soon died.

*Post Mortem.* Some turgescence in the vessels of the pia mater. Opposite the middle of the right parietal bone, there was, in the cortical substance of the cerebrum, a blackish spot, about the size of a shilling, which appeared mashed or bruised, and its organization entirely destroyed. No vestige of fissure or fracture in any part of the cranial parietes; no effusion of blood or water. This then is a case of *concussion;* but what difference would a *fracture* of the skull have made?

*Cure of Whitlow by Compression.* Dr. Balfour has extended *pressure* to the cure of deep seated inflammation of the aponeurotic and subjacent tissues of the fingers. In the *Medical Journal* for March, he relates several cases of whitlow cured by *mechanically* unloading the inflamed vessels through the medium of pressure. As we are far from believing that inflammation consists in mere *mechanical distention* of the blood-vessels, so we cannot readily concur in the Doctor's proposal for *mechanical* contraction of them. We know, indeed, that there is no more common cause of inflammation than pressure, *ab externo.* The Doctor, however, will doubtless prosecute the practice farther, and state the results.

*Spina Bifida.* Dr. Pliny Hales [*Lond. Med. Journ.* 229], relates the case of a child affected with spina bifida. The tumour was punctured on the 18th day, and its contents, a limpid fluid, partially discharged. The operation was reiterated frequently, the wounds always healing readily. Irritation of the internal surface of the sac by a needle brought on no inflammation, and compression with a pad effected no adhesion. The little patient eventually exhibited symptoms of hydrocephalus, and died on the 62d day. The alvine evacuations had uniformly been green, inclining to black. On *dissection*, the posterior portion of the canal in the lumbar vertebræ was entirely wanting. Below the lumbar vertebræ the dura matral sheath of the spinal marrow was merely protected by the common integuments. Here it formed a sac, containing the cauda equina and the fluid above-mentioned. There was much water in the ventricles of the brain.

*Syphilis.* Assistant Surgeon Boyle [*Med. Chirurg. Journal* 27], draws the attention of the Medical Public towards a quicker mode of curing syphilitic ulcers of the genitals, than has yet been tried; namely, that of suddenly impregnating the system with calomel, in *scruple doses*, whereby the patient is generally cured in ten or fifteen days, and without any violent ptyalism, or hypercatharsis. Mr. Cunningham, of H. M. S. Rochefort, has long been in the habit of curing recent chancres in this way; and in our next Periscope, we shall return to the subject.

